# High-fat but not sucrose intake is essential for induction of dyslipidemia and non-alcoholic steatohepatitis in guinea pigs

**DOI:** 10.1186/s12986-016-0110-1

**Published:** 2016-08-09

**Authors:** David Højland Ipsen, Pernille Tveden-Nyborg, Bidda Rolin, Günaj Rakipovski, Maria Beck, Line Winther Mortensen, Lasse Færk, Peter Mikael Helweg Heegaard, Peter Møller, Jens Lykkesfeldt

**Affiliations:** 1 Department of Veterinary Disease Biology, Faculty of Health and Medical Sciences, University of Copenhagen, Ridebanevej 9, 1870 Frederiksberg C, Denmark; 2 Diabetes Pharmacology, Global research, Novo Nordisk, Novo Nordisk Park 1, 2760 Måløv, Denmark; 3 National Veterinary Institute, Technical University of Denmark, Bülowsvej 27, 1870 Frederiksberg C, Denmark; 4 Department of Public Health, Section of Environmental Health, University of Copenhagen, Øster Farimagsgade 5A, 1353 Copenhagen, Denmark

**Keywords:** Non-alcoholic fatty liver disease, Non-alcoholic steatohepatitis, Dyslipidemia, High-fat diet, Sucrose, Guinea pigs, Cholesterol

## Abstract

**Background:**

Non-alcoholic fatty liver disease (NAFLD) and dyslipidemia are closely related. Diet plays an important role in the progression of these diseases, but the role of specific dietary components is not completely understood. Therefore, we investigated the role of dietary sucrose and fat/cholesterol on the development of dyslipidemia and NAFLD.

**Methods:**

Seventy female guinea pigs were block-randomized (based on weight) into five groups and fed a normal chow diet (control: 4 % fat), a very high-sucrose diet (vHS: 4 % fat, 25 % sucrose), a high-fat diet (HF: 20 % fat, 0.35 % cholesterol), a high-fat/high-sucrose diet (HFHS: 20 % fat, 15 % sucrose, 0.35 % cholesterol) or a high-fat/very high-sucrose diet (HFvHS: 20 % fat, 25 % sucrose, 0.35 % cholesterol) for 16 and 25 weeks.

**Results:**

All three high-fat diets induced dyslipidemia with increased concentrations of plasma cholesterol (*p* < 0.0001), LDL-C (*p* < 0.0001) and VLDL-C (*p* < 0.05) compared to control and vHS. Contrary to this, plasma triglycerides were increased in control and vHS compared to high-fat fed animals (*p* < 0.01), while circulating levels of free fatty acids were even between groups. Histological evaluation of liver sections revealed non-alcoholic steatohepatitis (NASH) with progressive inflammation and bridging fibrosis in high-fat fed animals. Accordingly, hepatic triglycerides (*p* < 0.05) and cholesterol (*p* < 0.0001) was increased alongside elevated levels of alanine and aspartate aminotransferase (*p* < 0.01) compared to control and vHS.

**Conclusion:**

Collectively, our results suggest that intake of fat and cholesterol, but not sucrose, are the main factors driving the development and progression of dyslipidemia and NAFLD/NASH.

**Electronic supplementary material:**

The online version of this article (doi:10.1186/s12986-016-0110-1) contains supplementary material, which is available to authorized users.

## Background

Non-alcoholic fatty liver disease (NAFLD) is the most common chronic liver disease in the Western world [1] and is closely associated with dyslipidemia [2, 3]. Affecting more than 30 % of the general adult population and with the potential to progress from simple steatosis to irreversible and life-threatening non-alcoholic steatohepatitis (NASH), it is an important public health concern [1, 4]. Disease progression is instigated by a series of parallel hits such as inflammation and oxidative stress, causing hepatocyte damage (e.g. metabolic dysfunction, DNA injury and apoptosis) and irreversible fibrosis, ultimately leading to cirrhosis and liver failure [5]. Although the etiology of NAFLD is not yet fully elucidated, changes in food composition are believed to play an essential role in disease progression [4]. Diets rich in saturated fat, cholesterol and non-complex carbohydrates (e.g. the disaccharide: sucrose) have been shown to induce dyslipidemia and hepatic lipid accumulation and are suggested to play a key role in the development of NASH in human patients [6–9]. However, potential interactions of different dietary components and whether certain components are more likely to cause NAFLD has not been determined [10]. Fat and cholesterol promotes oxidative stress, hepatocellular apoptosis, steatosis and NASH [5]. Furthermore, previous results from our group have indicated that sucrose may affect the development of NAFLD in guinea pigs [11]. Guinea pigs are one of the few species carrying the majority of their cholesterol in low density lipoprotein (LDL) particles; hence exhibiting a lipoprotein profile similar to that of humans [12–14]. Furthermore, we and others have reported that guinea pigs subjected to long-term feeding with diets high in fat, cholesterol and sucrose develop hepatic steatosis in accordance with NAFLD [11, 15, 16].

In the present study, we investigated the specific effects of dietary sucrose and fat/cholesterol, alone and in combination, on the development of dyslipidemia and NAFLD or NASH.

## Methods

### Animals and experimental design

Seventy female Hartley guinea pigs, 10 weeks old (Charles River Laboratories, Lyon, France), were block-randomized (based on body weight (BW)) into five homologous groups (*n* = 14) following one week of acclimation. The animals were group-housed in floor pens with wood shavings, hay, straw and environmental enrichment. Food and water was provided ad libitum and a 12 h light–dark cycle with temperatures between 20–24 °C was maintained. Groups were fed either chow (control), or chow-based diets of very high-sugar (vHS), high-fat (HF), high-fat/high-sugar (HFHS) or high-fat/very high-sugar (HFvHS) diets (Ssniff Spezialdiäten GmbH, Soest, Germany) (Table 1). The diets were stored at −20 °C and freshly thawed twice weekly (complete dietary compositions are shown Additional file 1).

**Table 1:** Composition of diets

Nutrient (g/kg diet)	Control	vHS	HF	HFHS	HFvHS
Protein	168	168	168	167	168
Fat	42	43	200	200	199
Carbohydrates (total)	471	535	363	379	411
Cholesterol	–	–	3.5	3.5	3.5
Sucrose (total amount added to the diet)	–	250	–	150	250
Metabolizable energy (MJ/kg)	12.3	13.4	16.4	16.7	17.2

Food intake in each group was estimated daily by weighing feed-remains prior to refill. After either 16 or 25 weeks, guinea pigs were semi-fasted over-night (no feed, but access to hay), pre-anaeshetized with 0.08 ml/kg BW Zoletil-mix, placed on isoflurane and euthanized by decapitation following an intra-cardial blood sample as previously described [11, 17]. Organs were rapidly collected, rinsed in phosphate buffered saline, weighed and stored at −80 °C or in paraformaldehyde for histological examinations.

### Oral glucose tolerance test

An oral glucose tolerance test (OGTT) was performed after 15 and 24 weeks. Guinea pigs were semi-fasted overnight and dosed orally with a 50 % glucose solution (Amgros I/S, Copenhagen, Denmark) by syringe (2 g glucose/kg BW). Blood glucose was measured with an Accu-Chek Aviva glucometer (Roche A/S Diagnostics, Basel, Switzerland) in triplicate or duplicates at time points 0, 15, 30, 45, 90, 120 and 180 min post-glucose consumption.

### Plasma samples

All samples obtained at euthanasia were collected intra-cardially, whereas samples taken during the study period (baseline triglyceride (TG) and total cholesterol (TC)) were collected from the *vena saphena* [18]. Samples for alkaline phosphatase (ALP) and free fatty acids (FFA) were collected in heparin and NaF-coated microvettes (Sarstedt, Nümbrecht, Germany), respectively. Alanine aminotransferase (ALT), aspartate aminotransferase (AST), TG and TC were collected in K_3_EDTA-coated microvettes (Sarstedt, Nümbrecht, Germany). Blood samples for all other analyses were collected in a K_3_EDTA-flushed 10 ml syringe. The analyses of ALP, AST, ALT, FFA, TG and baseline TC were performed on a Cobas 6000 (Roche Diagnostic Systems, Berne, Switzerland) according to manufacturer’s specifications. Lipoprotein fractions (very low density lipoprotein (VLDL), LDL and high density lipoprotein (HDL)) alongside TC at week 16 and 25 were analyzed by the *Lipoprotein Analysis Laboratory* (Wake Forest School of Medicine, Winston-Salem, North Carolina, USA) as described previously [19]. Serum amyloid A was determined by ELISA (Tridelta Development Ltd, Phase SAA Assay, Kildare, Ireland) and expressed as μg/ml porcine SAA equivalents as previously described [20].

### Liver samples

TC and TG were analyzed on liver homogenates sampled from the left lateral (*lobus hepatis sinister lateralis*) and right medial (*lobus hepatis dexter medialis*) lobes on a Cobas 6000 according to manufacturer’s specifications and as previously described [11].

### Telomere length

The average telomere length was measured from total genomic DNA from liver tissue using real-time quantitative PCR as described previously [21, 22]. For measurement of telomere repeat copies (T), the primers were: telg- 5′-CGG TTT GTT TGG GTT TGG GTT TGG GTT TGG GTT TGG GTT-3′ and telc- 5′-GGC TTG CCT TAC CCT TAC CCT TAC CCT TAC CCT TAC CCT-3′. Cycling conditions were: 2 min at 50 °C, 2 min of 95 °C, followed by 2 cycles of 95 °C for 15 s, 52 °C for 15 s and 36 cycles of 95 °C for 15 s, 62 °C for 15 s and 71 °C for 15 s. For measurement of single copy gene (S), the primers were: globin- 5′- ACT GGT CTA GGA CCC GAG AAG-3′ and globin- 5′- TCA ATG GTG CCT CTG GAG ATT-3′. The PCR was carried out in a 384-well 7900HT FAST Real-Time PCR System (Applied Biosystems, Slangerup, Denmark) using a reaction mix with 10–20 ng of genomic DNA in 1× SYBR® Green PCR Master Mix (Applied Biosystems, Slangerup, Denmark). The results are reported as the relative telomere length, i.e. the ratio of telomere repeat copy number (T) to single copy gene (S) copy number (T/S ratio).

### Single cell gel electrophoresis (comet) assay

DNA strand breaks were measured as previously described [23]. Strand breaks were visually scored and assigned to one of five classes in a blinded fashion as described by [23]. Cells treated with Ro19-8022 (gift from F. Hoffmann-La Roche, Basel, Switzerland) and white light were used as controls. The level of DNA damage was expressed as a total score calculated as:

$$ \left( Number\  of\  class\ I \cdot 1+ Number\  of\  class\ II \cdot 2 + Number\  of\  class\ III \cdot 3+ Number\  of\  class\ IV \cdot 4 + Number\  of\  class\ V \cdot 5\right)- total\  number\  of\  scored\  comets $$

### Histology

Paraformaldehyde fixed sections of the left lateral liver lobe were imbedded in paraffin cut into 2–4 μm cross-sections and stained with Mayer’s Haematoxylin and Eosin (H&E) or Masson’s trichrome stain as previously described [11]. All histological evaluations were performed in a blinded fashion. Sections were evaluated by scoring three lobuli, defined by the presence of at least two portal areas surrounding a central vein, and in accordance with the semi-quantitative scoring scheme suggested by Kleiner et al. [24] as follows: Steatosis was graded from 0–3 reflecting the amount of lipids: 0: <5 %; 1: 5–33 %; 2: >33–66 %; and 3: >66 %. Lobular inflammation was evaluated as the number of inflammatory foci (defined as at least three inflammatory cells in close proximity of each other) in a ×200 field as 0: no foci; 1: <2 foci per field; 2: 2–4 foci per field; 3: >4 foci or diffuse infiltration of the entire field. Portal inflammation was scored as 0: none to minimal, 1: greater than minimal. The presence of ballooning hepatocytes were acknowledged as 0: none; 1: few (but definite ballooning hepatocytes); or 2: many ballooning hepatocytes. Fibrosis was evaluated on entire sections stained by Masson’s trichrome. Fibrosis was graded as: 0: not present; 1: perisinusoidal *or* periportal; 1A: mild, zone 3 perisinusoidal; 1B: moderate, zone 3 perisinusoidal; 1C: portal/periportal; 2: perisinusoidal *and* portal/periportal; 3: bridging fibrosis; 4: cirrhosis.

### Statistical analysis

All statistical analyses were performed in SAS Enterprice Guide 7.1 (SAS Institute Inc, Cary, North Carolina, USA) and graphs were made in GraphPad Prism 6.06 (GraphPad Software, La Jolla, California, USA). Weight, plasma TG and TC were analyzed by a generalized linear mixed model with random effect of animals. The rest of the data was analyzed using a generalized linear model and presented as means with standard deviations (SD). Data with inhomogeneous variance was logarithmically transformed and then analyzed. Subsequently, data was back-transformed and presented as geometric means with 95 % confidence intervals. Tukey’s multiple comparisons test was used in all cases. Ordinal data (histopathological liver scores), DNA damage scores and telomere lengths were analyzed using non-parametric statistics followed by Bonferroni post-hoc test and are presented as medians with range. A p-value below 0.05 was considered statistically significant.

## Results

### Dietary regimes and OGTT

**Fig. 1:**
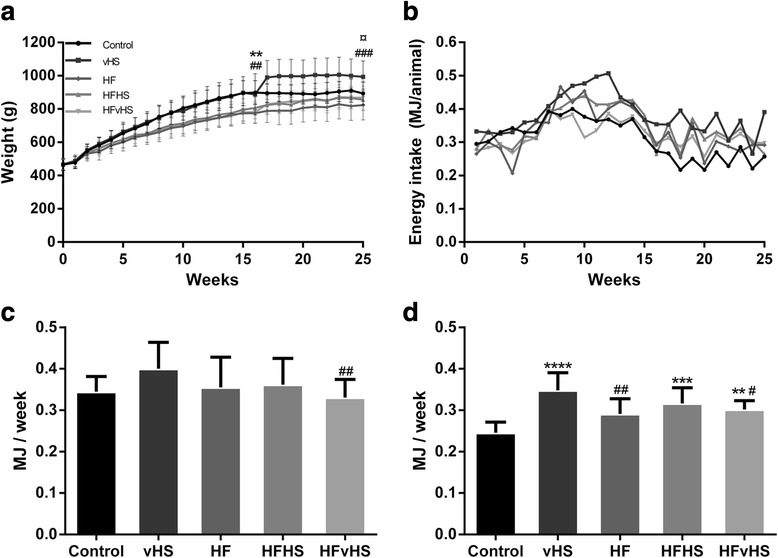
Weight and energy intake during the study period. **a** Control and vHS animals weighed more than high-fat fed animals after 16 weeks. vHS continued to weigh more compared to high-fat fed animals after 25 weeks, while control only weighed more than HF. Average weekly energy intake (**b**) and weekly cumulative energy intake per group until week 16 (**c**) and 25 (**d**) suggest that vHS had greater energy intake compared to the other groups. Means with SD, *n* = 7–14. HFD vs. Control: **** *p* < 0.0001 *** *p* < 0.001 ** *p* < 0.01. HFD vs. vHS: ### *p* < 0.001 ## *p* < 0.01 # *p* < 0.05. HF vs. Control: ¤ *p* < 0.05

There were no differences in BW between groups at study start. Control and vHS gained more weight resulting in significantly higher BW compared to the high-fat diet (HFD) groups (HF, HFHS and HFvHS) after 16 weeks (*p* < 0.01) (Fig. 1a). After 25 weeks, BW only differed between control and HF (*p* < 0.05) and vHS and HFD (*p* < 0.001). Initially, average energy intake increased in all groups, after which they declined and then remained stable throughout the study period (Fig. 1b). Cumulative weekly energy intake was higher in vHS compared to HFvHS (*p* < 0.01), but similar among other groups, after 16 weeks (Fig. 1c). After 25 weeks, cumulative energy intake was higher in vHS (*p* < 0.0001), HFHS (*p* < 0.001) and HFvHS (*p* < 0.01) compared to control and increased in vHS compared to HF (*p* < 0.01) and HFvHS (*p* < 0.05) (Fig. 1d). No difference between dietary regimes and glucose tolerance was recorded at either time-point (Additional file 2).

### Dyslipidemia and inflammation

**Fig. 2:**
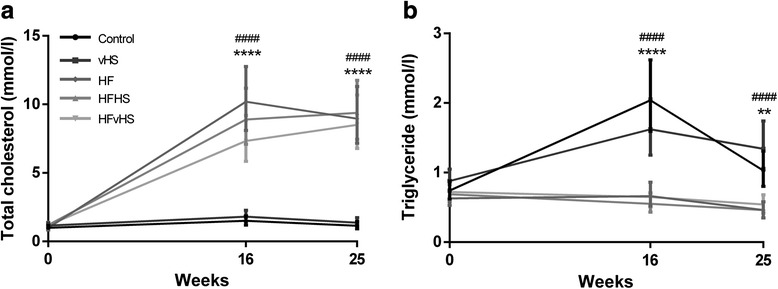
Plasma total cholesterol and triglycerides. After 16 and 25 weeks, total cholesterol (**a**) was significantly increased by high-fat diets, while triglycerides (**b**) increased in control and vHS. Geometric means with 95 % confidence interval, *n* = 7–14. HFD vs. Control: **** *p* < 0.0001 ** *p* < 0.01. HFD vs. vHS: #### *p* < 0.0001

Plasma TC and TG (Fig. 2a and b) did not differ between groups at baseline. At 16 and 25 weeks, plasma TC was increased in HF, HFHS and HFvHS compared to control and vHS (*p* < 0.0001). Contrary to this, plasma TG was elevated after 16 weeks (*p* < 0.0001) and 25 weeks (*p* < 0.01) in control and vHS compared to HFD. FFA did not differ between any groups at any time point. After 16 weeks on the diets, VLDL-C (*p* < 0.05) and LDL-C (*p* < 0.0001) concentrations were increased in HFD groups compared to control and vHS (Table 2). The dyslipidemia persisted after 25 weeks, i.e. increased VLDL-C (*p* < 0.001) and LDL-C (*p* < 0.0001) in HFD groups compared to control and vHS (Table 3). While HDL-C increased upon high-fat feeding, this was not statistically significant relative to control and vHS after 16 or 25 weeks. SAA concentrations were lower in HF (*p* < 0.01) and HFvHS (*p* < 0.001) compared to control and lower in all HFD groups compared to vHS (*p* < 0.01) after 16 weeks (Table 2). At 25 weeks, only HFvHS displayed lower SAA compared to vHS (*p* < 0.01) (Table 3).

**Table 2:** Circulating lipids, relative liver weight and biochemical markers after 16 weeks on diets

	Control	vHS	HF	HFHS	HFvHS
VLDL-C (mM) ^a^	0.19 (0.11–0.33)	0.18 (0.11–0.30)	1.01 (0.60–1.72) ^** ###^	0.89 (0.52–1.51) ^** ##^	0.68 (0.40–1.16) ^* #^
LDL-C (mM) ^a^	1.22 (0.92–1.62)	1.55 (1.17–2.06)	9.04 (6.81–12.0) ^**** ####^	7.86 (5.90–10.4) ^**** ####^	6.54 (4.93–8.68) ^**** ####^
HDL-C (mM) ^a^	0.04 (0.03–0.06)	0.04 (0.03–0.06)	0.07 (0.04–0.10)	0.10 (0.06–0.15)	0.09 (0.06–0.15)
FFA (mM)	0.77 ± 0.23	0.68 ± 0.16	0.50 ± 0.15	0.51 ± 0.09	0.52 ± 0.21
SAA (μg/mL) ^a^	89.4 (47.5–168)	182 (91.8–359)	14.3 (7.22–28.3) ^** ####^	22.5 (10.7–47.5) ^##^	10.8 (5.77–20.4) ^*** ####^
% Liver weight ^a^	2.16 (1.92–2.43)	2.29 (2.03–2.57)	4.72 (4.19–5.31) ^**** ####^	4.63 (4.11–5.21) ^**** ####^	4.30 (3.82–4.84) ^**** ####^
ALT (U/L) ^a^	43.1 (34.1–54.7)	32.4 (25.6–41.1)	92.5 (73.0–117) ^*** ####^	106 (83.8–128) ^****^^####^	98.1 (76.0–127)^*** ####^
AST (U/L) ^a^	67.0 (47.3–95.2)	51.5 (36.3–73.1)	403 (284–572) ^**** ####^	450 (317–639) ^**** ####^	323 (221–471) ^**** ####^
ALP (U/L)	70.3 ± 5.53	60.7 ± 18.5	59.0 ± 14.6	53.9 ± 9.19	51.9 ± 17.3

*n* = 7. Mean with SD. Compared to control **** *p* < 0.0001*** *p* < 0.001 ** *p* < 0.01 * *p* < 0.05. Compared to vHS #### *p* < 0.0001### *p* < 0.001 ## *p* < 0.01 # *p* < 0.05

^a^ Data analysis performed on log10 transformed data, presented as geometric means with 95 % confidence interval

**Table 3:** Circulating lipids, relative liver weight and biochemical markers after 25 weeks on diets

	Control	vHS	HF	HFHS	HFvHS
VLDL-C (mM) ^a^	0.12 (0.07–0.20)	0.13 (0.08–0.23)	1.02 (0.60–1.74) ^**** ####^	1.38 (0.81–2.35) ^**** ####^	0.89 (0.52–1.51) ^**** ###^
LDL-C (mM) ^a^	0.99 (0.74–1.31)	1.15 (0.87–1.52)	7.74 (5.83–10.3) ^**** ####^	7.83 (5.90–10.4) ^**** ####^	7.38 (5.56–9.79) ^**** ####^
HDL-C (mM) ^a^	0.03 (0.02–0.05)	0.04 (0.02–0.06)	0.06 (0.04–0.10)	0.08 (0.05–0.12)	0.07 (0.04–0.10)
FFA (mM)	0.48 ± 0.22	0.61 ± 0.12	0.58 ± 0.13	0.64 ± 0.13	0.43 ± 0.17
SAA (μg/mL) ^a^	46.6 (24.8–87.7)	88.3 (46.9–166)	20.9 (10.6–41.3)	48.1 (24.3–95.2)	12.3 (6.56–23.2) ^##^
% Liver weight ^a^	2.07 (1.84–2.32)	2.55 (2.27–2.87)	4.90 (4.35–5.51) ^**** ####^	5.19 (4.61–5.84) ^**** ####^	4.97 (4.42–5.59)^**** ####^
ALT (U/L) ^a^	37.0 (29.2–46.9)	30.2 (23.8–38.2)	77.7 (61.3–98.3) ^** ####^	101 (79.7–128) ^**** ####^	86.4 (68.3–109) ^*** ####^
AST (U/L) ^a^	43.6 (30.7–61.8)	43.7 (30.8–62.0)	259 (183–368) ^**** ####^	428 (302–607) ^**** ####^	445 (314–631) ^**** ####^
ALP (U/L)	60.1 ± 4.88	59.6 ± 13.1	45.9 ± 5.18	43.3 ± 1.70	47.0 ± 7.05

*n* = 7. Mean with SD. Compared to control **** *p* < 0.0001*** *p* < 0.001 ** *p* < 0.01. Compared to vHS #### *p* < 0.0001 ### *p* < 0.001

^a^ Data analysis performed on log10 transformed data, presented as geometric means with 95 % confidence interval

### Liver biochemistry and genomic damage

**Fig. 3:**
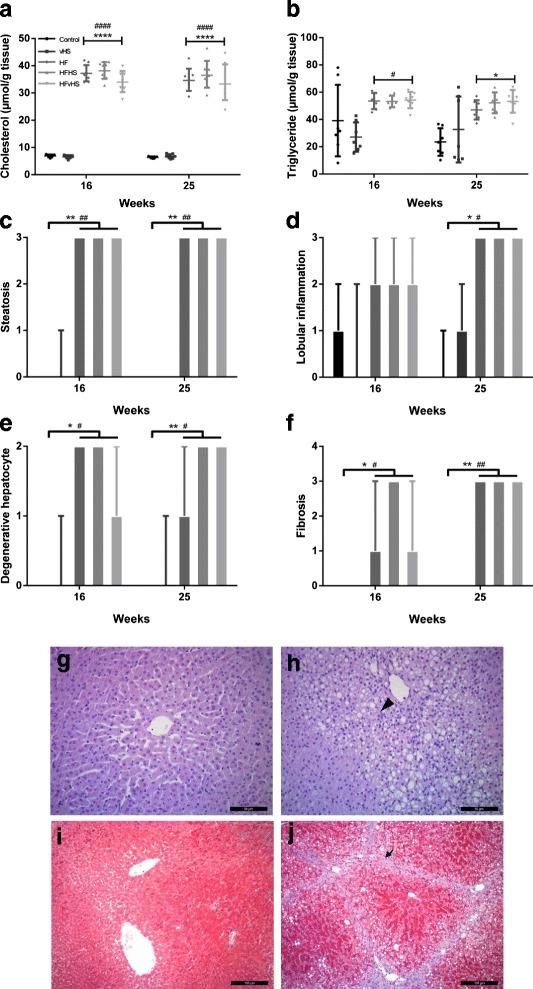
Biochemical and histological characteristics of livers. Hepatic lipid content (**a** and **b**), histological scoring of liver sections in accordance to [24] (**c**-**f**) and representative liver sections for control/vHS (**g** and **i**) and HFD (**h** and **j**). The arrow head and arrow indicates inflammatory cells and fibrosis, respectively. Hepatic cholesterol was increased by high-fat feeding at 16 and 25 weeks (**a**), while hepatic triglycerides were significantly increased in HFD groups after 25 weeks compared to control (**b**). Compared to control and vHS, HFD groups had grade 3 hepatic steatosis after 16 weeks, which persisted until the end of the study at 25 weeks (**c**, **g** and **h**). Similarly, high-fat diet induced progressive inflammation (**d**), hepatocyte ballooning (**e**) and fibrosis (**f**, **i** and **j**). Geometric means with 95 % confidence interval (**a**), means with SD (**b**) and medians with range (**c**-**f**). Scale bar 50 μm (**f** and **g**) and 100 μm (**h** and **i**), *n* = 6–7 (HFvHS *n* = 6 for histology at week 16 due to technical difficulties). HFD vs. Control: **** *p* < 0.0001 ** *p* < 0.01 * *p* < 0.05. HFD vs. vHS: #### *p* < 0.0001 ## *p* < 0.01 # *p* < 0.05

Compared to control and vHS, liver weight relative to BW increased upon high-fat feeding after 16 and 25 weeks (*p* < 0.0001) (Table 2 and 3). Similar results were observed for absolute liver weights (Additional file 3), substantiating that increased relative liver weights were not caused by lower BW in HFD groups. Accordingly, lipids were increased in the liver of the high-fat fed animals: Hepatic TC was increased in the HF, HFHS and HFvHS compared to both control and vHS (*p* < 0.0001) on both time-points (Fig. 3a). After 16 weeks hepatic TG was increased in the HFD groups compared to vHS (*p* < 0.05). After 25 weeks, an increase in hepatic TG was seen compared to control (*p* < 0.05), but not when compared to vHS animals (Fig. 3b). Compared to controls and vHS, plasma ALT (*p* < 0.001) and AST (*p* < 0.0001) were increased in all HFD groups at 16 weeks (Table 2) and remained elevated after 25 weeks (ALT *p* < 0.01, AST *p* < 0.0001) (Table 3). Plasma ALP did not differ between any groups at any time point (*p* > 0.05). Genomic damage as assessed by the length of telomeres and level of DNA strand breaks did not differ between groups (*p* > 0.05) (Additional file 4).

### Histology

Hepatic steatosis (grade 3), evident as micro- and macrovesicular lipid accumulation in zone 3, was found in most HFD animals, but not in control and vHS animals after 16 and 25 weeks (*p* < 0.01) (Fig. 3c, g and h). At week 16, lobular inflammation was not significantly higher in HFD groups compared to vHS. However, hepatic inflammation continued to progress in all HFD groups, resulting in severe inflammation (grade 3), which was significantly higher compared to control and vHS at week 25 (*p* < 0.05) (Fig. 3d). Concurrently, hepatocyte ballooning was more prominent in HFD groups at both time-points compared to control and vHS (*p* < 0.05) (Fig. 3e). Fibrosis (grade 1–3) was significantly increased in all HFD groups compared to control and vHS at week 16 (*p* < 0.05). At week 25, fibrosis, bridging between central veins (grade 3), was seen in almost all HFD animals, while fibrosis was absent in control and vHS (*p* < 0.01) (Fig. 3f, i and j). Portal inflammation was absent in all groups at both time points, apart from a single control animal at 16 weeks. The exact histological scoring is provided in Additional file 5.

## Discussion

The present study shows that dietary fat and cholesterol, but not sucrose, are the main factors driving the progression of dyslipidemia and NAFLD to NASH in a guinea pig model. Additionally, adding sucrose to a high-fat diet does not exacerbate the metabolic or hepatic consequences of a high-fat diet *per se*.

Expectedly, addition of cholesterol to the dietary regime increased circulating levels of TC, similar to findings of other studies utilizing cholesterol-rich [25, 26] or high-fat diet [27]. Though Plasma TC and TG concentrations were not affected by addition of sucrose consistent with results from mice and rats fed sucrose at levels of 32–35 % of total caloric intake [28, 29], circulating levels of TC and TG upon sucrose feeding have also been reported [30–33] It is possible that sucrose induced dyslipidemia differs mechanistically from the high fat induced [31], and moreover very high levels (≥60 %) of sucrose are applied to induce dyslipidemia [31–33]. Thus, it is possible that higher dietary sucrose concentrations may have been necessary to promote dyslipidemia in the current study; indeed calories originating from sucrose and fat were not equal potentially confounding the effect of sucrose. However, the translational relevance of models utilizing extremely high levels of sucrose have been questioned [34]. Consequently, the levels of dietary sucrose in this study may have more relevance to human consumption, albeit still being high.

Decreased levels of hepatic LDL-receptors and concomitant increased levels of circulating TC and LDL-C has been reported in guinea pigs subjected to a high-fat diet [35]. Accordingly, we found that LDL-C and VLDL-C increased upon high-fat feeding, regardless of dietary content of sucrose. This contradicts previous findings of a sucrose imposed elevation of TC and LDL-C when added to a high-fat diet in male guinea pigs [13, 36]. The observed dissimilarity may be due to differences in dietary composition as the latter high-fat diets did not contain excess cholesterol [13, 36] and/or a gender associated effect, as female rats—in contrast to males—proved resistant to sucrose-induced hypertriglyceridemia [37]. In agreement with our findings, circulating levels of lipids did not increase in humans placed on eucaloric diets, consuming 20 % of calories as sucrose for 10 weeks [38].

BW increased in all groups over time, but high-fat fed animals do not become obese and compared to the control and vHS groups, this is in accordance with previous results by us and others [11, 13, 16, 35]. Lack of comparable weight gain despite similar caloric intake might partly be due to hepatic lipid accumulation, rendering lipids unavailable for other tissues. After 16 weeks, HFHS and HFvHS increased energy-intake compared to control and consequently weight differences were eliminated between HFHS, HFvHS and control. Compared to control, HF also tended to increase energy-intake, but not enough to completely eliminate the weight difference. The apparent increase in BW observed for vHS after week 16 was due to the randomization procedure: animals were randomized, but not block-randomized based on weight at euthanasia and by chance, most of the animals with the highest BW were randomly chosen to continue on the diet. Regardless, vHS did not develop dyslipidemia or NAFLD. Thus, weight differences are not thought to influence the results of this study, and animals exposed to either vHS or high-fat diet were not obese compared to controls.

FFA release increases with increasing fat mass in humans [39] and the lack of increased FFA concentrations is likely to reflect the absence of obesity as reported in a non-obese rabbit-model of NASH [40]. Furthermore, plasma TG was not increased in response to the high-fat diet. This could be due to increased TG clearance from the blood and/or decreased hepatic TG production. Accordingly, guinea pigs on high-fat diet (25.1 % fat) displayed reduced plasma TG compared to their low-fat fed counterparts [13]. Lipoprotein lipase activity was increased by high-fat feeding, most likely contributing to the decreased circulating levels of TG [13]. Similarly, plasma TG was also lower in rats fed a high-fat, high-cholesterol diet compared to chow or a high-fat diet without cholesterol [41]. In these rats, hepatic microsomal triglyceride transfer protein mRNA expression was suppressed, potentially limiting hepatic VLDL-TG production [41]. Indeed, hepatic TG production may be compromised as NAFLD progresses from simple steatosis towards steatohepatitis. In humans, NASH is associated with impaired VLDL synthesis and secretion and reduced apoB100 synthesis [42, 43]. Hence, hepatic retention of TG, limiting TG availability for storage in adipose tissue, may constitute a causal mechanism in the progression of NASH in the non-obese phenotype of the dyslipidemic guinea pig model.

Guinea pig SAA - a systemic marker of inflammation [20] - was not induced by high-fat feeding at any of the two time points, similar to results from high fat fed (15 % fat, 1.35 % cholesterol) mice [44]. Our findings may suggest that systemic inflammation is not prominent in this model, at least when assessed by systemic SAA level. Alternatively, it could be speculated that low SSA levels was due to reduced liver function, supported by histopathology and increased ALT and AST levels, rendering the liver unable to produce and/or secrete SAA.

Our study revealed hepatocyte ballooning after 16 weeks of high-fat feeding, signifying the presence of NASH and distinguishing it from simple steatosis [45, 46]. This is further supported by the recorded inflammatory foci and progression of fibrosis in the HFD groups. While hepatic fibrosis is not necessary for the diagnosis of NASH, it represents a critical step in the progression of the disease, setting the stage for further liver damage such as cirrhosis and hepatocellular carcinoma [41]. However, high-fat diet models often induce mild hepatic fibrosis while rarely leading to severe progressive fibrosis [47, 48], except in guinea pigs [11]. After 16 weeks, guinea pigs in the HF and HFvHS group exhibited mild fibrosis, while the HFHS group already displayed bridging fibrosis (grade 3). After 25 weeks, all three HFD groups had formed bridging fibrosis. Consistent with our findings of circulating levels of lipids, sucrose feeding alone did not induce NAFLD, nor did it affect hepatic outcomes when added to a high-fat diet. Contrary to our results, 60–70 % sucrose promoted development of hepatic steatosis in both rats [49–51] and mice [52, 53]. However, while levels of hepatic inflammatory cytokines were increased [49], hepatic triglycerides were not significantly elevated [49, 51]. Thus, sucrose is seemingly not able to induce NASH.

Diseases characterized by chronic tissue regeneration, such as cirrhosis ensuing from progressive NASH, ultimately results in telomere shortening [54]. This promotes genomic instability paralleled by DNA strand damage which may constitute an underlying disease aspect playing an important role in NAFLD, especially with regards to fibrosis progression [54]. However, DNA strand breaks and telomere length was not different between groups. Consequently, these do not seem to be underlying mechanisms of NAFLD and NASH in this particular animal model. The null results on hepatic DNA damage is in keeping with earlier results in rats showing no altered levels of DNA strand breaks after feeding with saturated fats [55] and sucrose [56–59]. Alternatively, livers may have to become cirrhotic before notable telomere shortening can be detected, which may also explain the absence of telomere shortenings.

## Conclusion

Dietary sucrose alone or in combination with a high-fat diet did not affect the development of dyslipidemia or NASH. Thus, disease development appears to be driven mainly by dietary fat and cholesterol, but the current study is not able to distinguish between effects of dietary fat and cholesterol. Furthermore, the present diets contained high levels of saturated fatty acids and extrapolation of the results to dietary regimes differing in fatty acid composition and content should be done with caution. However, encompassing a similar histopathology indicates that the model may closely resemble the human condition. Based on the systemic and hepatic changes observed, our findings may reiterate the idea of fat and cholesterol as critical dietary factors with regards to disease progression.

## Abbreviations

ALP, alkaline phosphatase; ALT, alanine aminotransferase; AST, aspartate aminotransferase; BW, body weight; FFA, free fatty acids; H&E, Mayer’s haematoxylin and eosin; HDL-C, high density lipoprotein cholesterol; HF, high-fat; HFD, high-fat diet; HFHS, high-fat high-sucrose; HFvHS, high-fat very high-sucrose; LDL-C, low density lipoprotein cholesterol; NAFLD, non-alcoholic fatty liver disease; NASH, non-alcoholic steatohepatitis; OGTT, oral glucose tolerance test; SD, standard deviation; T/S ratio, ratio of telomere repeat copy number (T) to single copy gene (S) copy number; TC, total cholesterol; TG, triglycerides; vHS, very high-sucrose; VLDL-C, very low density lipoprotein cholesterol

## Additional files

Additional file 1: Exact dietary composition. The complete dietary composition including fatty acid composition. *Vitamin & trace element content (addition per kg feed): 25.0 IU Vitamin A (E672), 1.50 IU, Vitamin D3 (E671), 0.125 g Vitamin E (all-rac-alpha-tocopherylacetate) (3a700), 0.08 g Vitamin K3 (MNB), 0.08 g Vitamin B1 (Thiamine mononitrate), 0.03 g Vitamin B2 (Riboflavin), 0.05 g Ca Pantothenate, 0.025 g Vitamin B6 (pyridoxol hydrochloride) (3a831), 0.00015 g Vitamin B12 (Cyanocobalamine), 0.09 g Niacin, 0.009 g Folic acid, 0.0005 g Biotin, 0.100 g Inositol, 0.100 g Iron (II)-sulfate monohydrate (E1), 0.005 Copper (II)-sulfate pentahydrate (E4), 0.03 g Manganese (II)-sulfate monohydrate (E5), 0.002 g Cobalt (II)-carbonate monohydrate (E3), 0.05 g Zinc sulfate monohydrate (E6), 0.002 g Calcium iodate anhydrate (E2), 0.0001 g Sodium selenite (E8). ** 1.00 g NaCl added to HFvHS as soybean isolate contains approximately 1.5 % NaCl). (DOCX 17 kb) Additional file 2: Oral glucose tolerance tests. High-fat diets did not induce glucose intolerance as shown by oral glucose tolerance tests conducted after 15 (A) and 24 weeks (B). Means with SD, *n* = 7. HFD vs. Control: **** *p* < 0.0001 ** *p* < 0.01 * *p* < 0.05. HFD vs. vHS: #### *p* < 0.0001 ### *p* < 0.001 # *p* < 0.05. Control vs. vHS: θθθθ *p* < 0.0001 θθθ *p* < 0.001 θ *p* < 0.05. (TIF 497 kb) Additional file 3: Absolute liver weight. The absolute liver weights were increased in HFD compared to Control and vHS after 16 and 25 weeks. Means with SD, *n* = 7. HFD vs. Control: **** *p* < 0.0001. HFD vs. vHS: ### *p* < 0.001. HFD vs. vHS: ## *p* < 0.01. (TIF 211 kb) Additional file 4: Hepatic telomere length and DNA strand breaks. T/S expresses the ratio of the mean telomere repeat copies (T) to a reference single copy gene (S) and did not differ between groups (A). Additionally, the extent of DNA damage, measured as strand breaks, did not differ between groups (B). Medians with range, *n* = 5–7. (TIF 442 kb) Additional file 5: Frequencies of hepatic steatosis, lobular inflammation, ballooning hepatocytes and fibrosis. Histopathological scoring of hepatic steatosis, lobular inflammation, ballooning (degenerative) hepatocytes and fibrosis done according to Kleiner et al. (20). Scores are listed as 16 weeks | 25 weeks (HFvHS *n* = 6 for fibrosis scoring at week 16 due to technical difficulties). (DOCX 15 kb)
