# Supplementary material for: High-fat but not sucrose intake is essential for induction of dyslipidemia and non-alcoholic steatohepatitis in guinea pigs
Source: Nutr Metab (Lond). 2016 Aug 9;13:51. doi: 10.1186/s12986-016-0110-1 (PMC4979160; doi:10.1186/s12986-016-0110-1)
Supplement: Additional file 1: — Exact dietary composition. The complete dietary composition including fatty acid composition. *Vitamin & trace element content (addition per kg feed): 25.0 IU Vitamin A (E672), 1.50 IU, Vitamin D3 (E671), 0.125 g Vitamin E (all-rac-alpha-tocopherylacetate) (3a700), 0.08 g Vitamin K3 (MNB), 0.08 g Vitamin B1 (Thiamine mononitrate), 0.03 g Vitamin B2 (Riboflavin), 0.05 g Ca Pantothenate, 0.025 g Vitamin B6 (pyridoxol hydrochloride) (3a831), 0.00015 g Vitamin B12 (Cyanocobalamine), 0.09 g Niacin, 0.009 g Folic acid, 0.0005 g Biotin, 0.100 g Inositol, 0.100 g Iron (II)-sulfate monohydrate (E1), 0.005 Copper (II)-sulfate pentahydrate (E4), 0.03 g Manganese (II)-sulfate monohydrate (E5), 0.002 g Cobalt (II)-carbonate monohydrate (E3), 0.05 g Zinc sulfate monohydrate (E6), 0.002 g Calcium iodate anhydrate (E2), 0.0001 g Sodium selenite (E8). ** 1.00 g NaCl added to HFvHS as soybean isolate contains approximately 1.5 % NaCl). (DOCX 17 kb) [file 12986_2016_110_MOESM1_ESM.docx]

**Additional File 1** Exact dietary composition

| **Nutrients (g/kg diet)** | **Control** | **vHS** | **HF** | **HFHS** | **HFvHS** |
| --- | --- | --- | --- | --- | --- |
| **Alfalfa** | 220 | 220 | 220 | 220 | 220 |
| **Wheat** | 283 | 185 | 290 | 103 | 27.0 |
| **Barley** | 180 | - | - | - | - |
| **Sucrose** | - | 250 | - | 150 | 250 |
| **Cellulose (lignocellulose)** | 46.0 | 44.0 | 40.0 | 46.0 | 56.0 |
| **Sunflower meal** | 30.0 | 30.0 | 30.0 | 30.0 | 30.0 |
| **Soybean meal** | 120 | 40.0 | 70.0 | 60.0 | 30.0 |
| **Soybeans (full fat)** | 26.0 | 26.0 | 26.0 | 26.0 | 46.0 |
| **Soybean concentrate** | 20.0 | 120 | 80.0 | 120 | - |
| **Soybean isolate (90% protein)** | - | - | - | - | 10.8 |
| **Amino acids** | 5.00 | 5.50 | 5.90 | 5.00 | 5.50 |
| **Vitamins & trace element*** | 10.0 | 10.0 | 10.0 | 10.0 | 10.0 |
| **Vitamin C (Stay-C)** | 29.0 | 29.0 | 29.0 | 29.0 | 29.0 |
| **NaCl**** | 4.0 | 4.0 | 4.0 | 4.0 | 1.0 |
| **Calcium phosphate**  **(monobasic)** | 12.9 | 15.4 | 15.4 | 16.4 | 17.4 |
| **Calcium propionate** | 5.5 | 5.5 | 5.5 | 5.5 | 5.5 |
| **Calcium carbonate** | 2.5 | 1.5 | 1.5 | 1.5 | 1.5 |
| **Choline Cl** | 3.0 | 3.0 | 3.0 | 3.0 | 3.0 |
| **Sugar beet pulp** | 10.0 | 10.0 | 10.0 | 10.0 | 10.0 |
| **Cholesterol** | - | - | 3.50 | 3.50 | 3.50 |
| **Coconut oil, hydrogenated** | - | - | 180 | 180 | 180 |
| **Soybean oil** | 21.0 | 27.0 | 2.0 | 5.0 | 5.0 |
| **Crude protein** | 168 | 168 | 168 | 167 | 168 |
| **Crude fat** | 42 | 43 | 200 | 200 | 199 |
| **Crude fiber** | 126 | 113 | 113 | 114 | 113 |
| **Crude ash** | 65 | 65 | 65 | 66 | 58 |
| **Starch** | 279 | 127 | 189 | 77 | 29 |
| **Sugar** | 38 | 276 | 32 | 176 | 267 |
| **Carbohydrates** | 471 | 535 | 363 | 379 | 411 |
| **Fatty acids** |  |  |  |  |  |
| C 6:0 | - | - | 0.6 | 0.6 | 0.6 |
| C 8:0 | - | - | 9.9 | 9.9 | 9.9 |
| C 10:0 | - | - | 9.3 | 9.3 | 9.3 |
| C 12:0 | - | - | 81.9 | 81.9 | 81.9 |
| C 14:0 | 0.2 | 0.1 | 35.4 | 35.4 | 35.4 |
| C 16:0 | 6.3 | 5.9 | 22.9 | 22.7 | 22.6 |
| C 18:0 | 1.6 | 1.5 | 21.9 | 22.0 | 22.0 |
| C 20:0 | 0.2 | 0.1 | 0.3 | 0.3 | 0.3 |
| C 16:1 | 0.3 | 0.1 | 0.1 | 0.1 | 0.1 |
| C 18:1 | 8.7 | 9.4 | 5.3 | 5.6 | 5.5 |
| C 18:2 | 21.9 | 22.0 | 10.0 | 9.8 | 9.2 |
| C 18:3 | 3.7 | 3.5 | 2.3 | 2.2 | 2.1 |
