# Supplementary figures and images for: High-fat but not sucrose intake is essential for induction of dyslipidemia and non-alcoholic steatohepatitis in guinea pigs
Source: Nutr Metab (Lond). 2016 Aug 9;13:51. doi: 10.1186/s12986-016-0110-1 (PMC4979160; doi:10.1186/s12986-016-0110-1)

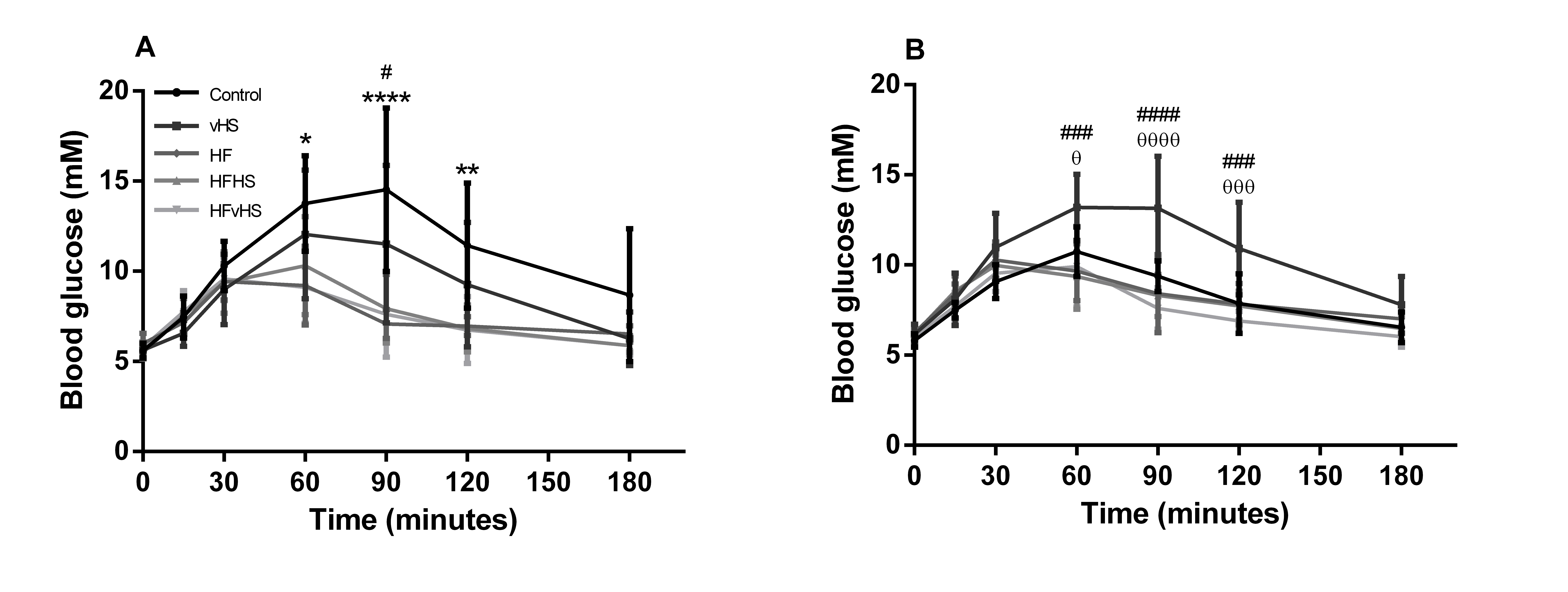

Supplement: Additional file 2: — Oral glucose tolerance tests. High-fat diets did not induce glucose intolerance as shown by oral glucose tolerance tests conducted after 15 (A) and 24 weeks (B). Means with SD, n = 7. HFD vs. Control: **** p < 0.0001 ** p < 0.01 * p < 0.05. HFD vs. vHS: #### p < 0.0001 ### p < 0.001 # p < 0.05. Control vs. vHS: θθθθ p < 0.0001 θθθ p < 0.001 θ p < 0.05. (TIF 497 kb) [file 12986_2016_110_MOESM2_ESM.tif]

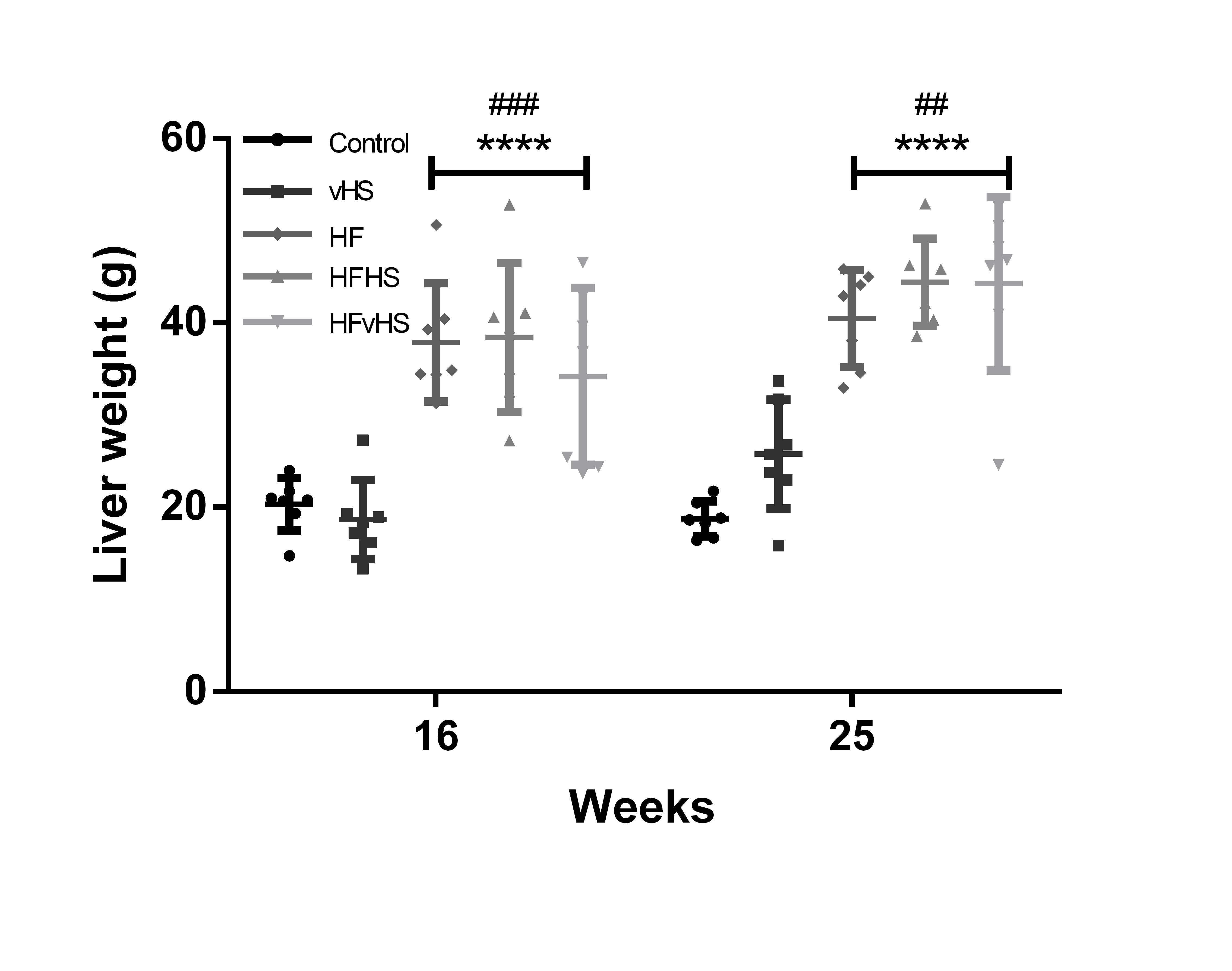

Supplement: Additional file 3: — Absolute liver weight. The absolute liver weights were increased in HFD compared to Control and vHS after 16 and 25 weeks. Means with SD, n = 7. HFD vs. Control: **** p < 0.0001. HFD vs. vHS: ### p < 0.001. HFD vs. vHS: ## p < 0.01. (TIF 211 kb) [file 12986_2016_110_MOESM3_ESM.tif]

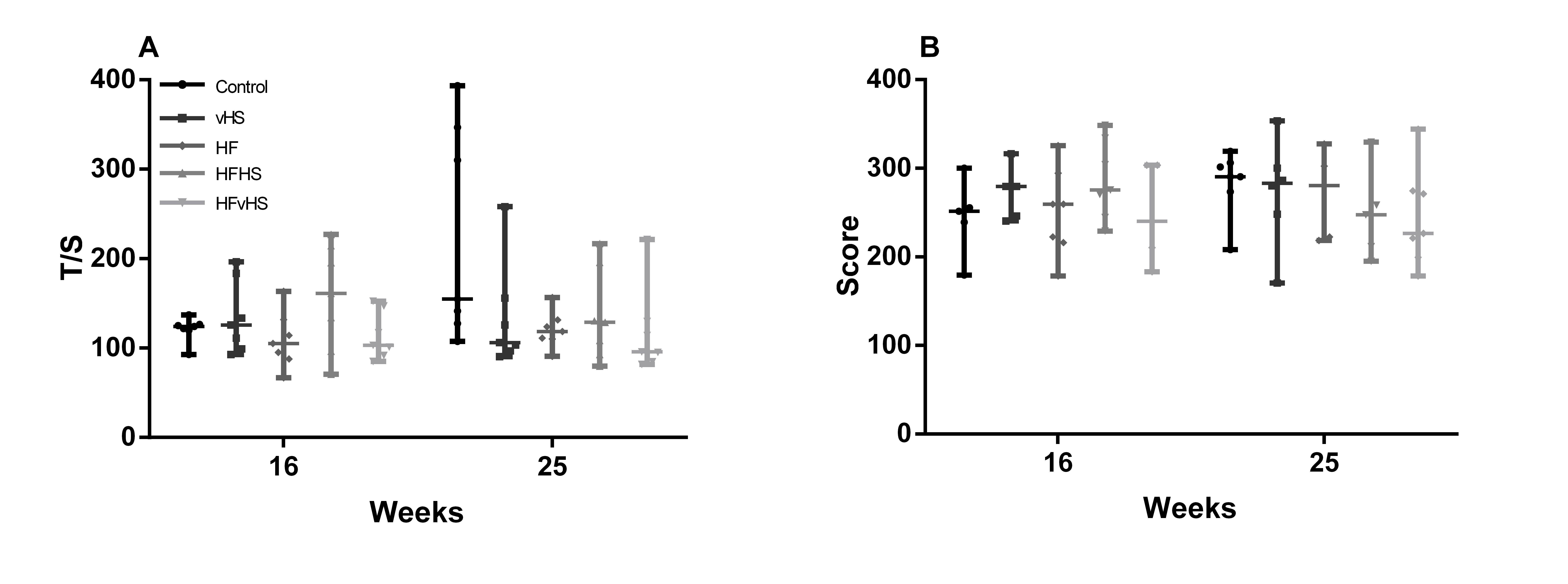

Supplement: Additional file 4: — Hepatic telomere length and DNA strand breaks. T/S expresses the ratio of the mean telomere repeat copies (T) to a reference single copy gene (S) and did not differ between groups (A). Additionally, the extent of DNA damage, measured as strand breaks, did not differ between groups (B). Medians with range, n = 5–7. (TIF 442 kb) [file 12986_2016_110_MOESM4_ESM.tif]
