# Supplementary material for: High-fat but not sucrose intake is essential for induction of dyslipidemia and non-alcoholic steatohepatitis in guinea pigs
Source: Nutr Metab (Lond). 2016 Aug 9;13:51. doi: 10.1186/s12986-016-0110-1 (PMC4979160; doi:10.1186/s12986-016-0110-1)
Supplement: Additional file 5: — Frequencies of hepatic steatosis, lobular inflammation, ballooning hepatocytes and fibrosis. Histopathological scoring of hepatic steatosis, lobular inflammation, ballooning (degenerative) hepatocytes and fibrosis done according to Kleiner et al. (20). Scores are listed as 16 weeks | 25 weeks (HFvHS n = 6 for fibrosis scoring at week 16 due to technical difficulties). (DOCX 15 kb) [file 12986_2016_110_MOESM5_ESM.docx]

**Additional file 5** Frequencies of hepatic steatosis, lobular inflammation, ballooning hepatocytes and fibrosis.

|  | | **Control** | **vHS** | **HF** | **HFHS** | **HFvHS** |
| --- | --- | --- | --- | --- | --- | --- |
| **Degree of steatosis** | | | | | | |
| **0** | 7/7 \| 7/7 | | 6/7 \| 7/7 | 0/7 \| 0/7 | 0/7 \| 0/7 | 0/7 \| 0/7 |
| **1** | 0/7 \| 0/7 | | 1/7 \| 0/7 | 0/7 \| 0/7 | 0/7 \| 0/7 | 0/7 \| 0/7 |
| **2** | 0/7 \| 0/7 | | 0/7 \| 0/7 | 0/7 \| 0/7 | 1/7 \| 0/7 | 2/7 \| 1/7 |
| **3** | 0/7 \| 0/7 | | 0/7 \| 0/7 | 7/7 \| 7/7 | 6/7 \| 7/7 | 5/7 \| 6/7 |
| **Lobular inflammation** | | | | | | |
| **0** | 2/7 \| 4/7 | | 5/7 \| 2/7 | 0/7 \| 0/7 | 1/7 \| 0/7 | 1/7 \| 0/7 |
| **1** | 3/7 \| 3/7 | | 1/7 \| 4/7 | 2/7 \| 0/7 | 0/7 \| 0/7 | 1/7 \| 1/7 |
| **2** | 2/7 \| 0/7 | | 1/7 \| 1/7 | 4/7 \| 0/7 | 3/7 \| 0/7 | 4/7 \| 0/7 |
| **3** | 0/7 \| 0/7 | | 0/7 \| 0/7 | 1/7 \| 7/7 | 3/7 \| 7/7 | 1/7 \| 6/7 |
| **Ballooning hepatocytes** | | | | | | |
| **0** | 7/7 \| 7/7 | | 6/7 \| 6/7 | 0/7 \| 0/7 | 0/7 \| 0/7 | 0/7 \| 0/7 |
| **1** | 0/7 \| 0/7 | | 1/7 \| 1/7 | 3/7 \| 5/7 | 3/7 \| 2/7 | 5/7 \| 3/7 |
| **2** | 0/7 \| 0/7 | | 0/7 \| 0/7 | 4/7 \| 2/7 | 4/7 \| 5/7 | 2/7 \| 4/7 |
| **Fibrosis** | | | | | | |
| **0** | 7/7 \| 7/7 | | 7/7 \| 7/7 | 0/7 \| 0/7 | 1/7 \| 0/7 | 1/6 \| 0/7 |
| **1** | 0/7 \| 0/7 | | 0/7 \| 0/7 | 0/7 \| 0/7 | 0/7 \| 0/7 | 0/6 \| 0/7 |
| **1A** | 0/7 \| 0/7 | | 0/7 \| 0/7 | 6/7 \| 1/7 | 1/7 \| 1/7 | 4/6 \| 2/7 |
| **1B** | 0/7 \| 0/7 | | 0/7 \| 0/7 | 0/7 \| 0/7 | 0/7 \| 0/7 | 0/6 \| 0/7 |
| **1C** | 0/7 \| 0/7 | | 0/7 \| 0/7 | 0/7 \| 0/7 | 0/7 \| 0/7 | 0/6 \| 0/7 |
| **2** | 0/7 \| 0/7 | | 0/7 \| 0/7 | 0/7 \| 0/7 | 0/7 \| 0/7 | 0/6 \| 0/7 |
| **3** | 0/7 \| 0/7 | | 0/7 \| 0/7 | 1/7 \| 6/7 | 5/7 \| 6/7 | 1/6 \| 5/7 |
| **4** | 0/7 \| 0/7 | | 0/7 \| 0/7 | 0/7 \| 0/7 | 0/7 \| 0/7 | 0/6 \| 0/7 |
